# Supplementary material for: Fast kernel-based association testing of non-linear genetic effects for biobank-scale data
Source: Nat Commun. 2023 Aug 15;14:4936. doi: 10.1038/s41467-023-40346-2 (PMC10427662; doi:10.1038/s41467-023-40346-2)
Supplement: Supplementary file 1 — Supplementary Information [file 41467_2023_40346_MOESM1_ESM.pdf]

Supplementary Figures

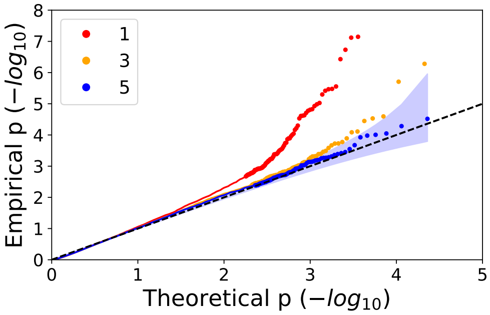

Supplementary Figure 1: **Accounting for linear effects in the application of FastKAST**. We regressed out linear effects in a window centered around the target window (superwindow). We varied the size of the the superwindow considering settings where the superwindow is equal to the target window and the superwindow contains three and five windows of equal physical length centered on the target window to find that a superwindow of size five leads to calibrated p-values. The phenotypes were simulated under a non-infinitesimal genetic architecture with heritability $h^2 = 0.5$, causal variant ratio $= 0.001$ and RARE distribution of causal variants. The two-sided 95% confidence interval was estimated using a beta distribution.

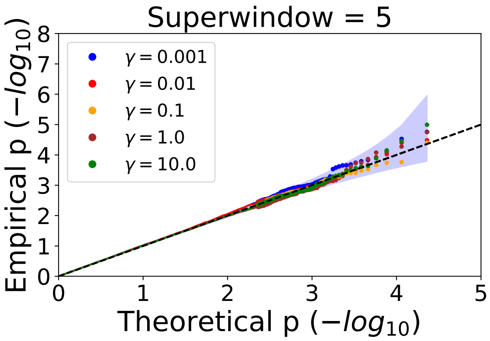

Supplementary Figure 2: **Calibration of FastKAST under the choice of specific values for the kernel hyperparameters**. We assessed the calibration of FastKAST with different values of the kernel hyperparameters under simulations with linear effects but no non-linear effect. We performed simulations with heritability $h^2 = 0.5$, causal variant ratio $= 0.001$, and RARE distribution of causal variants. We fit FastKAST, in turn, with RBF kernel hyperparameter: $(\gamma \in \{0.001, 0.01, 0.1, 1, 10\})$. The two-sided 95% confidence interval was estimated using a beta distribution.

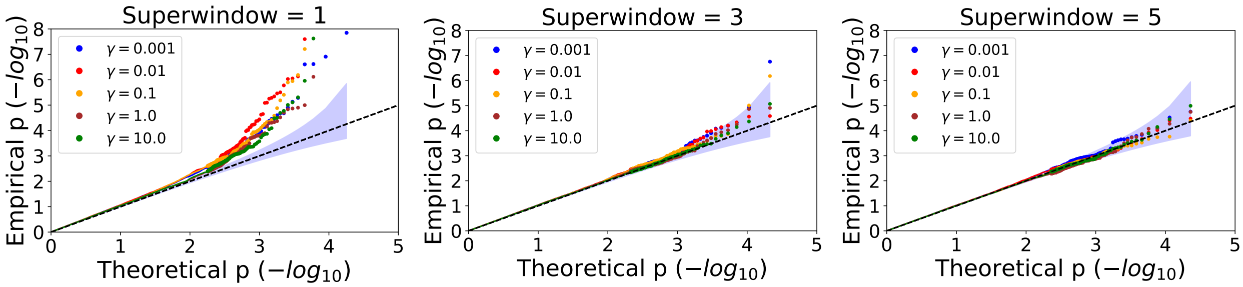

(a) (b) (c)

Supplementary Figure 3: **Accounting for linear effects in the application of FastKAST with a fixed hyperparameter**. We regressed out linear effects in a window centered around the target window (superwindow). We varied the size of the the superwindow considering settings where the superwindow is equal to the target window and the superwindow contains three and five windows of equal physical length centered on the target window to find that a superwindow of size five leads to calibrated p-values. We applied FastKAST with a fixed value of the hyperparameter γ. The architecture that we simulated is RARE with causal ratio = 0.001 and the MAF range of causal variants ranging from 0.01 to 0.05. The two-sided 95% confidence interval was estimated using a beta distribution.

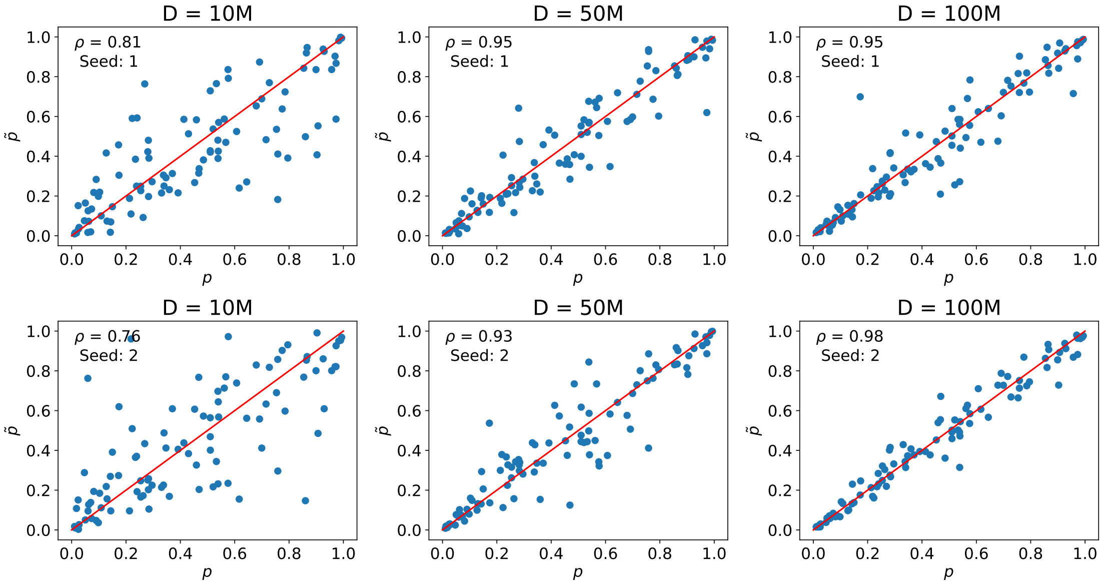

Supplementary Figure 4: **Correlation between p-values computed by FastKAST for varying values of the approximation dimension) and the true kernel on simulated phenotypes**. p represents the p-value computed using the exact kernel; $\tilde{p}$ represents the p-value computed by FastKAST. The experiments were conducted on traits simulated under a non-infinitesimal model setting (causal variant ratio=0.001 and MAF range: [0.009-0.011], with 100 random draws of fixed sized window with 100 kb. Each row represents results from a different random number seed.

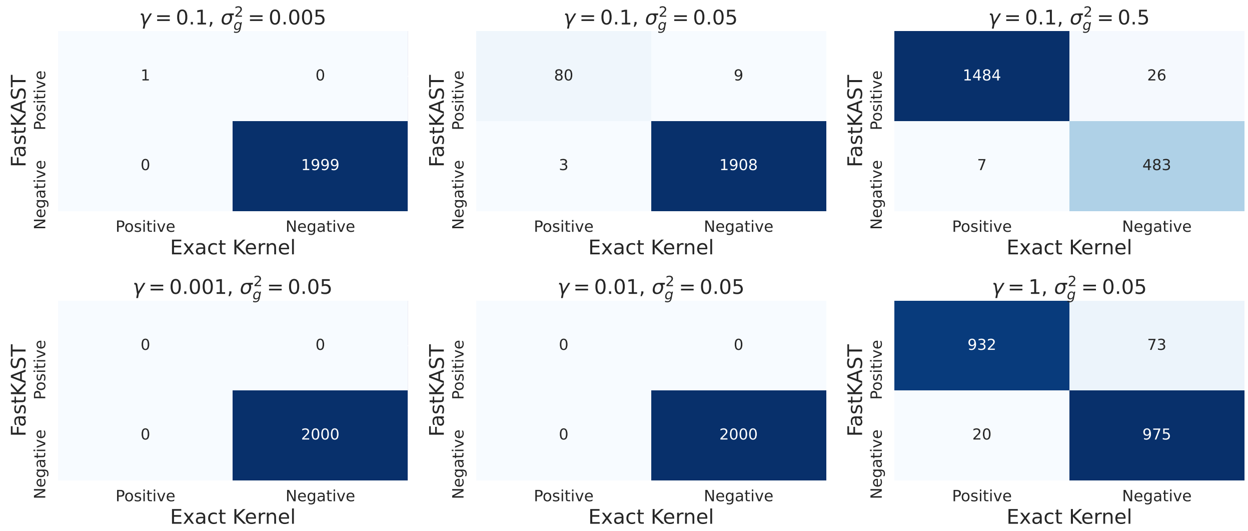

Supplementary Figure 5: **Comparison of the power of FastKAST and a test that uses the exact kernel**. We compared the power of FastKAST to an exact kernel under simulations described in the Section: **Power analysis of FastKAST**. TP denotes *True Positives* while FN denotes *False Negatives*. The first three settings correspond to a fixed value of the kernel hyperparameter γ while we vary the strength of the non-linear genetic effect σ^2. The next three settings explore varying kernel hyperparameters γ while the non-linear genetic effect σ^2 is set to 0.05. The significance threshold used is the same as that of the UKBB analysis: $\frac{0.05}{28,818}$, where 28,818 is the number of 100kb windows tested. For each method, "Positive" represents the number of tests that pass the significance threshold while "Negative" represents the number of the tests that do not pass the significance threshold. The percentage of tests where the two tests agree across each setting is: $(\gamma = 0.1, \sigma_g^2 = 0.005) : 100\%$; $(\gamma = 0.1, \sigma_g^2 = 0.05) : 99.4\%$; $(\gamma = 0.1, \sigma_g^2 = 0.5) : 98.35\%$; $(\gamma = 0.01, \sigma_g^2 = 0.05) : 100\%$; $(\gamma = 0.001, \sigma_g^2 = 0.05) : 100\%$; $(\gamma = 1, \sigma_g^2 = 0.05) : 95\%$.

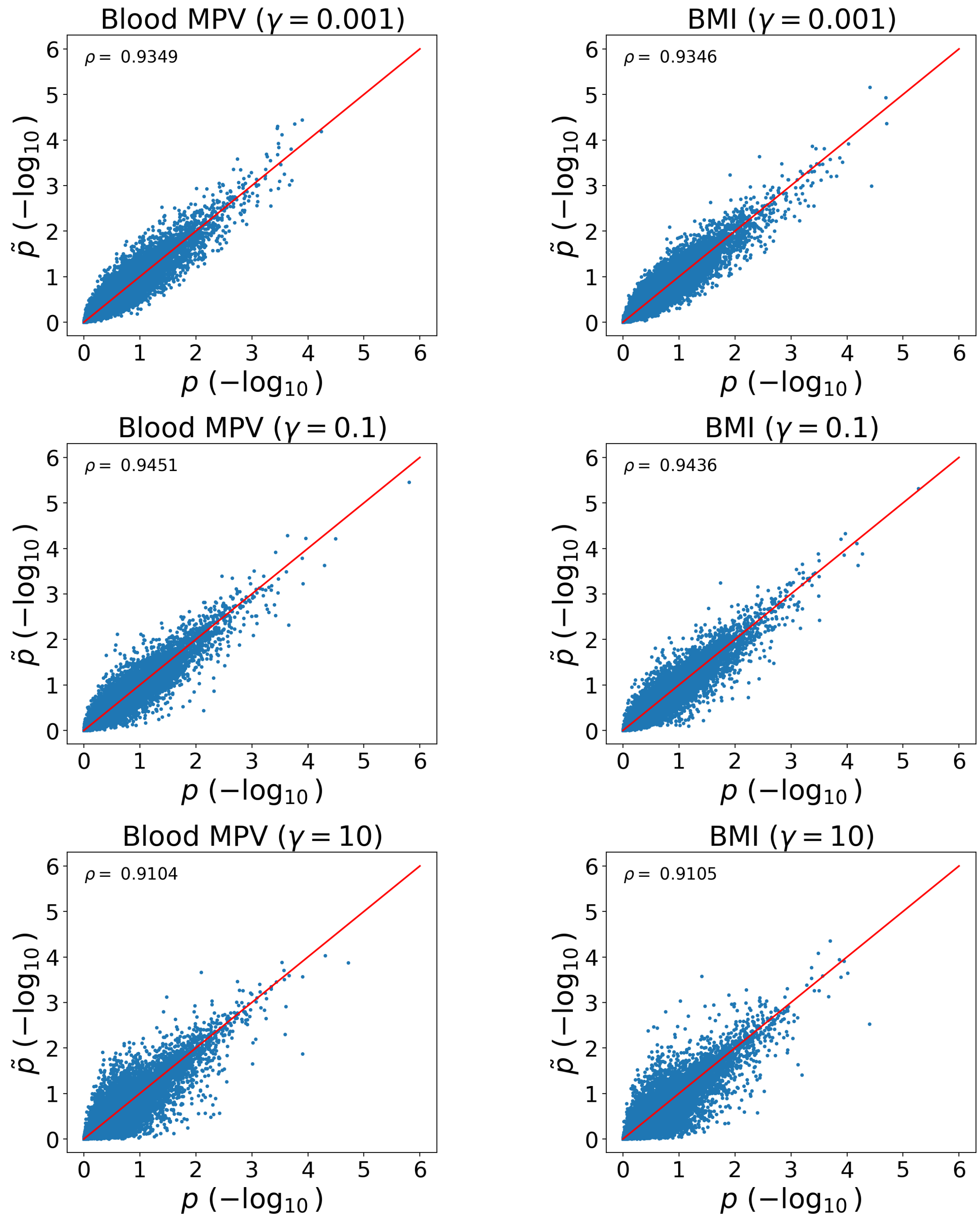

Supplementary Figure 6: **Correlation between the p-values computed by FastKAST and the true kernel on real phenotypes under various kernel hyperparameter values (γ).** We applied both methods to analyze non-overlapping 100 kb windows across $N = 5,000$ unrelated white British individuals in UKBB. FastKAST was applied using the default approximation dimension of $D = 50M$. p represents the p-value computed using the exact kernel; $\tilde{p}$ represents the p-value computed by FastKAST. In each figure, we report the Pearson correlation coefficient ρ between the two p-values across all windows.

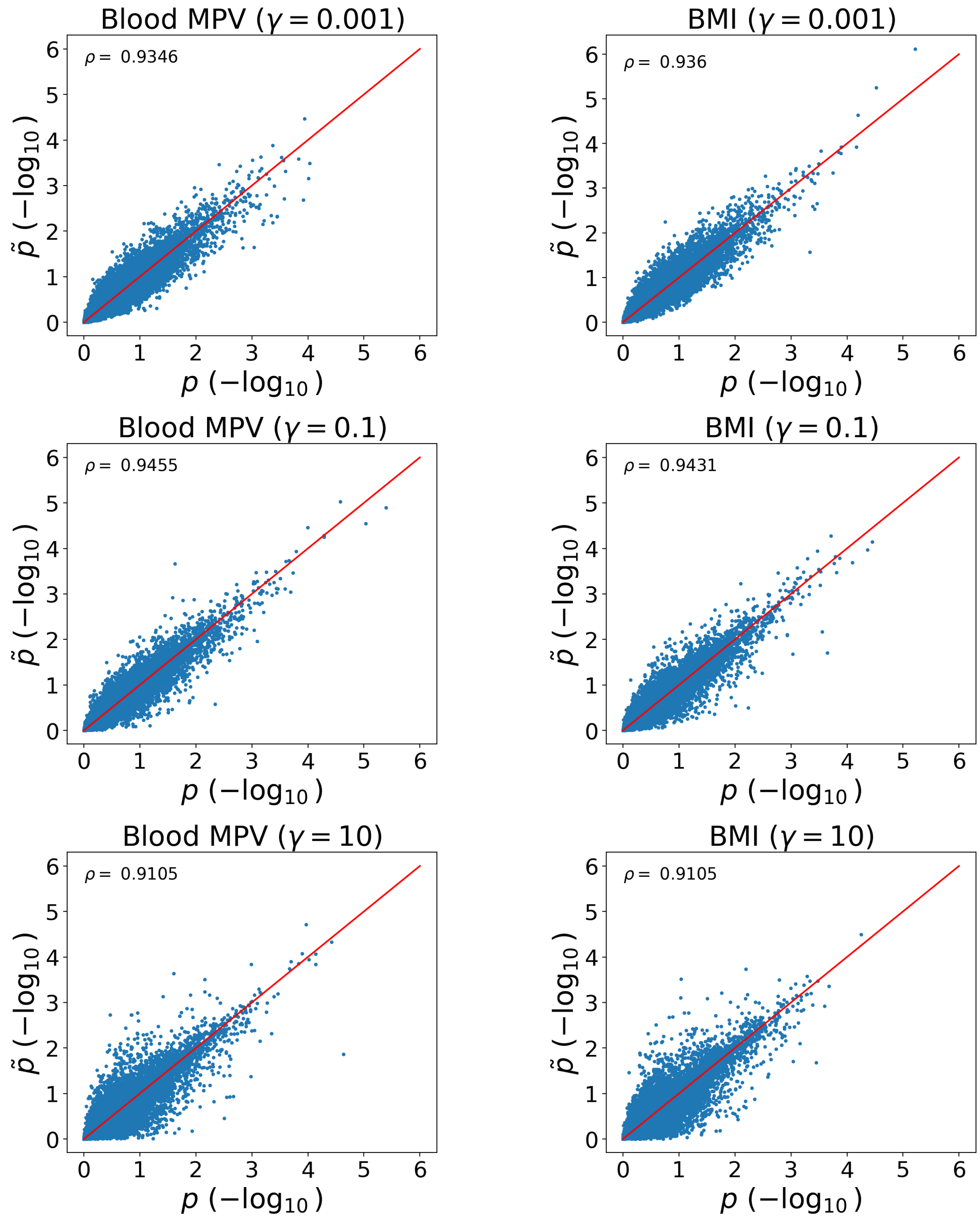

Supplementary Figure 7: **Correlation between p value from the approximate kernel and true kernel when analyzing a real trait phenotype under various kernel hyperparameter values** (γ). This is the same analysis as described in Supplementary Figure 6 but with a different random number seed. In each figure, we report the Pearson correlation coefficient ρ between the two p-values across all windows.

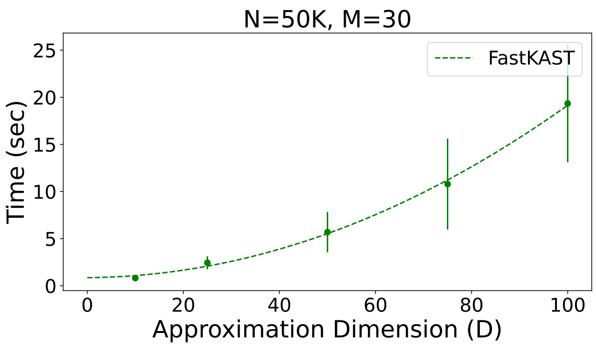

(a)

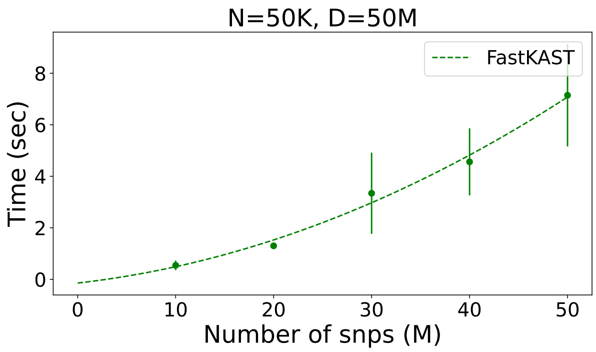

(b)

Supplementary Figure 8: **Computational efficiency of FastKAST**. (a) shows the runtime of FastKAST when increasing the approximation dimension D. (b) shows the runtime of FastKAST when increasing the number of SNPs M within the window. The figure only demonstrates the computational time of the singular value decomposition (SVD) which is the bottleneck of FastKAST. For each parameter setting, we report the mean (denoted as the dot) and the standard deviation (denoted as the error bars) of the runtime across 100 replicates.

Supplementary Tables

Supplementary Table 1: **False positive rate for varying p-value thresholds and genomic inflation factor**. We report the empirical type I error rate (false positive rate) of FastKAST at different p-value thresholds and the genomic inflation factor, assuming the hyperparameter is unknown. We report the false positive rate over 1.3 million tests from the simulation setting (ALL, Causal ratio=1). Among all tests, we do not observe any significance threshold at which the FPR is inflated significantly above the nominal level.

	α						λ_{gc}
Threshold	10^{-1}	10^{-2}	10^{-3}	10^{-4}	10^{-5}	10^{-6}	
FPR	0.99×10^{-1}	0.99×10^{-2}	0.98×10^{-3}	1.02×10^{-4}	0.92×10^{-5}	0.76×10^{-6}	0.99

Supplementary Table 2: **Robustness with respect to population structure (Number of principal components)**. We tested all the candidate trait-loci pairs that passed the prescreening threshold with various principal components. We report the p-value computed from FastKAST for the hyperparameter that attains the minimum p-value in our initial analysis for each locus.

Trait	Chr	Start (Mb)	End (Mb)	Number of PCs ($-log_{10}P$) 5	10	20	40
Alanine aminotransferase	22	44.3	44.4	12.50	12.50	12.57	12.51
Alkaline phosphatase	1	22.6	22.7	8.82	8.90	8.99	9.05
	6	24.1	24.2	9.78	9.76	9.85	9.83
	9	136.1	136.2	≥ 13	≥ 13	≥ 13	≥ 13
	9	136.2	136.3	11.03	11.00	11.03	11.06
Apolipoprotein B	19	19.3	19.4	≥ 13	≥ 13	≥ 13	≥ 13
	19	19.5	19.6	12.80	12.82	12.83	12.81
	19	19.6	19.7	≥ 13	≥ 13	≥ 13	≥ 13
	19	45.2	45.3	12.07	12.14	12.13	12.13
	19	45.3	45.4	≥ 13	≥ 13	≥ 13	≥ 13
	19	45.4	45.5	≥ 13	≥ 13	≥ 13	≥ 13
Aspartate aminotransferase	22	44.3	44.4	9.98	9.98	9.96	9.93
Body mass index	16	53.8	53.9	7.95	7.96	8.02	8.06
Cholesterol	19	19.6	19.7	12.26	12.29	12.35	12.33
Creatinine	1	15.8	15.9	9.94	9.94	9.92	9.93
Cystatin-C	20	23.9	24.0	≥ 13	≥ 13	≥ 13	≥ 13
Direct bilirubin	2	234.2	234.3	9.05	9.11	9.13	9.13
	2	234.3	234.4	≥ 13	≥ 13	≥ 13	≥ 13
	2	234.4	234.5	12.40	12.44	12.46	12.48
	2	234.5	234.6	≥ 13	≥ 13	≥ 13	≥ 13
	2	234.6	234.7	≥ 13	≥ 13	≥ 13	≥ 13
	2	234.9	235.0	≥ 13	≥ 13	≥ 13	≥ 13
Eosinophil count	11	57.1	57.2	11.29	11.30	11.29	11.30
HDL cholesterol	16	56.9	57.0	≥ 13	≥ 13	≥ 13	≥ 13
Hemoglobin A1c	6	20.6	20.7	8.58	8.60	8.69	8.74
LDL direct	19	19.3	19.4	9.11	9.13	9.17	9.15
	19	19.5	19.6	9.98	10.00	9.99	9.96
	19	19.6	19.7	≥ 13	≥ 13	≥ 13	≥ 13
Lipoprotein-A	6	159.8	159.9	9.83	9.91	9.88	9.86
	6	159.9	160.0	≥ 13	≥ 13	≥ 13	≥ 13
	6	160.0	160.1	≥ 13	≥ 13	≥ 13	≥ 13
	6	160.2	160.3	≥ 13	≥ 13	≥ 13	≥ 13
	6	160.4	160.5	≥ 13	≥ 13	≥ 13	≥ 13
	6	160.5	160.6	≥ 13	≥ 13	≥ 13	≥ 13
	6	160.6	160.7	≥ 13	≥ 13	≥ 13	≥ 13
	6	160.7	160.8	≥ 13	≥ 13	≥ 13	≥ 13
	6	160.9	161.0	≥ 13	≥ 13	≥ 13	≥ 13
	6	161.0	161.1	≥ 13	≥ 13	≥ 13	≥ 13
	6	161.1	161.2	≥ 13	≥ 13	≥ 13	≥ 13
	6	161.2	161.3	9.03	9.08	9.09	9.09
	6	161.3	161.4	≥ 13	≥ 13	≥ 13	≥ 13
	6	161.5	161.6	≥ 13	≥ 13	≥ 13	≥ 13
	6	161.6	161.7	≥ 13	≥ 13	≥ 13	≥ 13
	6	161.7	161.8	12.12	12.14	12.12	12.08
	6	161.8	161.9	≥ 13	≥ 13	≥ 13	≥ 13
Mean corpuscular hemoglobin	6	135.4	135.5	≥ 13	≥ 13	≥ 13	≥ 13
	16	0.1	0.2	≥ 13	≥ 13	≥ 13	≥ 13
Mean platelet volume	3	56.8	56.9	≥ 13	≥ 13	≥ 13	≥ 13
	12	122.3	122.4	9.44	9.44	9.44	9.42
Mean sphered cell volume	1	158.5	158.6	11.38	11.36	11.37	11.37
Monocyte count	22	17.6	17.7	10.59	10.60	10.63	10.62
Platelet count	3	56.8	56.9	12.23	12.20	12.21	12.21
Platelet distribution width	3	56.8	56.9	≥ 13	≥ 13	≥ 13	≥ 13
	20	57.5	57.6	≥ 13	≥ 13	≥ 13	≥ 13
RBC count	6	135.4	135.5	10.68	10.66	10.69	10.68
SHBG	1	107.5	107.6	9.29	9.29	9.25	9.27
Total bilirubin	2	234.1	234.2	9.59	9.60	9.66	9.65
	2	234.2	234.3	11.35	11.38	11.40	11.41
	2	234.3	234.4	≥ 13	≥ 13	≥ 13	≥ 13
	2	234.4	234.5	≥ 13	≥ 13	≥ 13	≥ 13
	2	234.5	234.6	≥ 13	≥ 13	≥ 13	≥ 13
	2	234.6	234.7	≥ 13	≥ 13	≥ 13	≥ 13
	2	234.7	234.8	9.94	9.91	9.90	9.84
	2	234.8	234.9	11.15	11.18	11.20	11.16
	2	234.9	235.0	≥ 13	≥ 13	≥ 13	≥ 13
	2	235.0	235.1	12.46	12.45	12.42	12.48
	11	2.9	3.0	9.32	9.31	9.33	9.39
Urate	1	15.8	15.9	11.05	11.08	10.91	10.92
	1	15.9	16.0	12.40	12.43	12.19	12.14
	4	9.9	10.0	≥ 13	≥ 13	≥ 13	≥ 13
	4	10.0	10.1	≥ 13	≥ 13	≥ 13	≥ 13
	4	10.1	10.2	≥ 13	≥ 13	≥ 13	≥ 13
	4	10.2	10.3	≥ 13	≥ 13	≥ 13	≥ 13
	4	10.3	10.4	≥ 13	≥ 13	≥ 13	≥ 13
Urea	1	155.1	155.2	8.57	8.57	8.50	8.53

Supplementary Table 3: **Loci with statistically significant non-linear effects ($p < 3.27 \times 10^{-8}$ accounting for the number of sets and traits tested)**. We report loci with p-values that are significant after regressing out linear effects of SNPs in five windows centered around the tested window (*Array Non-linear*, with a precision on $-\log_{10}$ p-value bounded by 13). We further report p-values after removing the quadratic effect of the current window in addition to the linear effect (*Array Non-linear + non-quadratic*, with a precision on $-\log_{10}$ p-value bounded by 13). Further, we report p-values after removing the linear effect when analyzing imputed genotypes in these windows (*Imputed Non-linear*, with a precision on $-\log_{10}$ p-value bounded by 13). We highlight loci which remain significant across all analyses with "***"; entries whose loci overlapped with those of the gene-set test are colored with grey background.

Trait	Chr	Start (Mb)	End (Mb)	Array ($-\log_{10}(p)$) Non-linear	Array ($-\log_{10}(p)$) Non-linear + non-quadratic	Imputed Non-linear ($-\log_{10}(p)$)
Alanine aminotransferase	22	44.3	44.4	12.08	0.51	2.87
Alkaline phosphatase	1	22.6	22.7	8.51	1.66	2.86
	6	24.1	24.2	9.37	0.58	0.4
	9	136.1	136.2	≥ 13	0.01	11.54
***	9	136.2	136.3	10.55	8.0	12.57
Apolipoprotein B	19	19.3	19.4	12.69	8.39	7.28
	19	19.5	19.6	12.35	1.74	4.0
	19	19.6	19.7	≥ 13	0.86	5.5
	19	45.2	45.3	11.65	1.75	0.72
	19	45.3	45.4	≥ 13	0.0	8.91
	19	45.4	45.5	≥ 13	0.04	2.24
Aspartate aminotransferase	22	44.3	44.4	9.48	0.08	2.74
Body mass index	16	53.8	53.9	7.54	0.08	3.29
Cholesterol	19	19.6	19.7	11.87	0.27	3.55
Creatinine	1	15.8	15.9	9.44	0.08	5.01
Cystatin-C	20	23.9	24.0	≥ 13	2.63	0.4
Direct bilirubin	2	234.2	234.3	8.66	9.27	1.8
	2	234.3	234.4	≥ 13	2.59	9.26
	2	234.4	234.5	11.98	4.61	≥ 13
	2	234.5	234.6	≥ 13	2.21	≥ 13
	2	234.6	234.7	≥ 13	0.67	≥ 13
	2	234.9	235.0	≥ 13	3.29	1.13
Eosinophil count	11	57.1	57.2	10.81	1.12	0.64
HDL cholesterol	16	56.9	57.0	≥ 13	1.52	0.55
Hemoglobin A1c	6	20.6	20.7	8.21	0.01	3.99
LDL direct	19	19.3	19.4	8.7	5.63	5.88
	19	19.5	19.6	9.52	1.92	2.36
	19	19.6	19.7	≥ 13	0.34	3.78
Lipoprotein-A	6	160.2	160.3	≥ 13	10.08	4.02
	6	160.4	160.5	≥ 13	≥ 13	5.52
	6	160.5	160.6	≥ 13	5.66	≥ 13
***	6	160.6	160.7	≥ 13	11.08	11.06
***	6	160.7	160.8	≥ 13	≥ 13	≥ 13
***	6	160.9	161.0	≥ 13	≥ 13	12.2
***	6	161.0	161.1	≥ 13	≥ 13	8.59
***	6	161.1	161.2	≥ 13	≥ 13	≥ 13
	6	161.2	161.3	8.62	10.8	5.4
	6	161.3	161.4	≥ 13	2.45	3.08
	6	161.5	161.6	≥ 13	9.45	3.06
***	6	161.6	161.7	≥ 13	≥ 13	9.71
	6	161.7	161.8	11.64	5.06	3.02
	6	161.8	161.9	≥ 13	1.28	4.28
	6	159.8	159.9	9.41	6.46	3.18
	6	159.9	160.0	≥ 13	5.87	4.44
	6	160.0	160.1	≥ 13	5.41	2.89
Mean corpuscular hemoglobin	6	135.4	135.5	≥ 13	0.41	8.1
	16	0.1	0.2	12.69	2.45	2.41
Mean platelet volume	3	56.8	56.9	≥ 13	1.17	10.38
	12	122.3	122.4	8.96	≥ 13	7.1
Mean sphered cell volume	1	158.5	158.6	10.89	2.99	1.49
Monocyte count	22	17.6	17.7	10.16	1.02	0.87
Platelet count	3	56.8	56.9	11.73	1.12	8.41
Platelet distribution width	3	56.8	56.9	≥ 13	0.84	≥ 13
	20	57.5	57.6	≥ 13	0.29	1.25
RBC count	6	135.4	135.5	10.22	0.34	5.83
SHBG	1	107.5	107.6	8.77	0.03	5.35
Total bilirubin	2	234.1	234.2	9.19	0.81	1.57
	2	234.2	234.3	10.92	≥ 13	4.15
	2	234.3	234.4	≥ 13	3.6	10.81
	2	234.4	234.5	≥ 13	6.78	≥ 13
	2	234.5	234.6	≥ 13	1.39	≥ 13
	2	234.6	234.7	≥ 13	0.57	≥ 13
	2	234.7	234.8	9.42	1.36	2.83
	2	234.8	234.9	10.73	1.83	4.92
	2	234.9	235.0	≥ 13	2.39	0.74
	2	235.0	235.1	11.94	2.09	2.91
	11	2.9	3.0	8.86	0.08	1.38
Urate	1	15.8	15.9	10.43	0.44	5.5
	1	15.9	16.0	11.71	≥ 13	2.76
	4	9.9	10.0	≥ 13	2.07	≥ 13
***	4	10.0	10.1	≥ 13	≥ 13	≥ 13
	4	10.1	10.2	≥ 13	5.83	3.4
	4	10.2	10.3	≥ 13	0.81	2.47
	4	10.3	10.4	≥ 13	0.03	10.17
Urea	1	155.1	155.2	8.02	0.08	7.82

Supplementary Table 4: **Protein-coding genes with statistically significant non-linear effects as detected by method ($p < 9.37 \times 10^{-8}$ accounting for the number of genes and traits tested)**. We reported genes with p-values that are significant after regressing out linear effects of SNPs in five windows centered around the tested window using FastKAST (denoted as $-log_{10}$Pval (FastKAST), the precision is bounded by 13; and we also reported the corresponding $-log_{10}$ p-value computed using SKAT (denoted as $-log_{10}$Pval (SKAT), the precision is bounded by 315). Highlighted rows indicate whether the corresponding significant trait-gene pair overlaps with the previous genome-wide analysis.

Trait	CHR	Gene	Start (Mb)	End (Mb)	$-log_{10}$Pval (FastKAST)	$-log_{10}$Pval (SKAT)
Alanine aminotransferase	22	*PNPLA3*	44.32	44.34	≥ 13	61.23
Alkaline phosphatase	9	*C9orf96*	136.24	136.27	≥ 13	282.96
Apolipoprotein B	19	*NCAN*	19.33	19.36	9.31	13.28
	19	*MAU2*	19.43	19.47	7.91	21.31
	19	*ZNF101*	19.78	19.79	10.33	8.20
	19	*BCAM*	45.31	45.32	≥ 13	132.82
	19	*PVRL2*	45.35	45.39	≥ 13	320.00
	19	*APOE*	45.41	45.41	≥ 13	320.00
Aspartate aminotransferase	22	*PNPLA3*	44.32	44.34	≥ 13	66.55
Creatinine	1	*CASP9*	15.82	15.85	8.62	0.06
	1	*DNAJC16*	15.86	15.89	8.45	1.52
Direct bilirubin	2	*SAG*	234.22	234.26	≥ 13	320.00
	2	*DGKD*	234.26	234.38	≥ 13	320.00
	2	*UGT1A8*	234.53	234.68	≥ 13	320.00
Eosinophil count	11	*TNKS1BP1*	57.07	57.09	≥ 13	2.37
	11	*PRG3*	57.14	57.15	≥ 13	5.69
HDL cholesterol	16	*CETP*	57.00	57.02	9.35	320.00
LDL direct	19	*APOE*	45.41	45.41	≥ 13	320.00
Lipoprotein-A	6	*FNDC1*	159.59	159.69	7.24	26.57
	6	*IGF2R*	160.39	160.53	≥ 13	320.00
	6	*SLC22A2*	160.64	160.68	7.06	248.48
	6	*SLC22A3*	160.77	160.87	≥ 13	320.00
	6	*LPA*	160.95	161.09	≥ 13	320.00
	6	*PLG*	161.12	161.17	12.68	295.99
	6	*AGPAT4*	161.56	161.65	≥ 13	320.00
Mean corpuscular hemoglobin	6	*SCGN*	25.65	25.70	8.54	34.88
	10	*MSMB*	51.55	51.56	7.4	1.26
Mean platelet volume	3	*ARHGEF3*	56.76	57.07	9.72	314.12
	12	*WDR66*	122.36	122.44	≥ 13	88.66
	20	*TUBB1*	57.59	57.60	≥ 13	141.04
Mean sphered cell volume	1	*OR10Z1*	158.58	158.58	9.07	11.10
	1	*SPTA1*	158.58	158.66	9.16	217.03
Platelet distribution width	12	*WDR66*	122.36	122.44	10.13	9.63
	20	*TUBB1*	57.59	57.60	≥ 13	320.00
	20	*EDN3*	57.88	57.90	11.53	20.29
RBC distribution width	19	*APOE*	45.41	45.41	10.59	93.73
SHBG	17	*DNAH2*	7.62	7.74	7.46	54.30
Total bilirubin	2	*SAG*	234.22	234.26	≥ 13	320.00
	2	*DGKD*	234.26	234.38	≥ 13	320.00
	2	*UGT1A8*	234.53	234.68	≥ 13	320.00
	11	*SLC22A18*	2.92	2.95	8.0	9.97
Urate	1	*CELA2B*	15.80	15.82	10.57	7.44
	1	*CASP9*	15.82	15.85	9.95	2.17
	1	*DNAJC16*	15.86	15.89	10.28	0.28
	4	*SLC2A9*	9.83	10.03	≥ 13	320.00
	4	*WDR1*	10.08	10.12	10.95	83.97
	4	*MEPE*	88.76	88.77	8.36	2.36
Urea	1	*MUC1*	155.16	155.16	7.47	2.69

Supplementary Table 5: **Comparison of SKAT and FastKAST on 53 quantitative traits in UKBB in the setting of general set-based association testing**. Here *Shared* represents signals detected by both SKAT and FastKAST for a given trait while *SKAT only* represents the SKAT exclusive signal, and *FastKAST only* represents the FastKAST exclusive signal. Here a signal is defined as a protein-coding genic region for which the p-value assigned by the corresponding method passes the Bonferroni correction threshold $p < 0.05/(10{,}078 \times 53)$.

Trait	Shared	SKAT only	FastKAST only
Age first birth	0	0	3
Alanine aminotransferase	33	8	85
Albumin	29	6	98
Alcohol intake frequency	0	0	16
Alkaline phosphatase	92	6	186
Apolipoprotein A	63	10	142
Apolipoprotein B	75	7	80
Aspartate aminotransferase	50	7	177
Basal metabolic rate	62	11	245
Direct bilirubin	21	8	29
Total bilirubin	29	4	74
Eosinophil count	94	15	237
Lymphocyte count	85	15	233
Mean corpuscular hemoglobin	149	27	244
Monocyte count	88	12	216
Mean platelet volume	232	23	304
Mean sphered cell volume	84	13	204
Platelet count	159	21	333
Platelet distribution width	121	19	255
RBC count	70	9	232
RBC distribution width	121	18	229
High light scatter reticulocyte count	109	10	218
White blood cell count	72	12	215
BMD Heel T-score	28	2	38
Body mass index	17	5	134
Diastolic blood pressure	8	3	61
Systolic blood pressure	5	2	53
C-reactive protein	50	8	94
Calcium	27	3	87
Cholesterol	72	7	96
Corneal Hysteresis	8	0	25
Creatinine	61	5	210
Creatinine in urine	0	0	1
Cystatin-C	50	6	232
FEV1-FVC ratio	37	6	152
FVC	24	10	199
Glucose	14	0	32
Hemoglobin A1c	103	8	239
HDL cholesterol	83	11	166
Height	256	27	607
IGF-1	76	9	221
LDL direct	62	9	68
Lipoprotein-A	15	3	4
Microalbumin in urine	1	0	0
Phosphate	30	1	54
Potassium in urine	0	0	4
SHBG	62	8	150
Sodium in urine	2	0	5
Testosterone	19	2	39
Total protein	53	9	155
Triglycerides	76	7	133
Urate	54	7	131
Urea	16	2	77

Supplementary Note 1

Proof of the sampling distribution of the approximate score statistic

Theorem 1. *Assuming that* $\boldsymbol{y} \sim \mathcal{N}(\boldsymbol{X\beta}, \sigma_g^2\boldsymbol{K} + \sigma_\epsilon^2\boldsymbol{I})$*, the approximate score statistic* $Q = \frac{1}{\sigma_\epsilon^2}\boldsymbol{y}^T\boldsymbol{P}\tilde{\boldsymbol{K}}\boldsymbol{P}\boldsymbol{y}$ *is distributed as a weighted sum of* χ_1^2 *variables under the null hypothesis* (H_0)*:*

$$\frac{1}{\sigma_\epsilon^2}\boldsymbol{y}^T\boldsymbol{P}\tilde{\boldsymbol{K}}\boldsymbol{P}\boldsymbol{y} \sim \sum_{n=1}^{N}\rho_n\chi_1^2$$

where ρ_n *denotes the* n^{th} *eigenvalue of the matrix* $\boldsymbol{P}\tilde{\boldsymbol{K}}\boldsymbol{P}$ *and* $\boldsymbol{P} = (\boldsymbol{I} - \boldsymbol{X}(\boldsymbol{X}^T\boldsymbol{X})^{-1}\boldsymbol{X}^T)$ *denotes the projection matrix.*

Proof. Under the null hypothesis $\sigma_g^2 = 0$, we have $\boldsymbol{y} \sim \mathcal{N}(\boldsymbol{X\beta}, \sigma_\epsilon^2\boldsymbol{I})$, therefore $\boldsymbol{Py} \sim \mathcal{N}(0, \sigma_\epsilon^2\boldsymbol{P})$. Since $\tilde{\boldsymbol{K}}$ is positive semi-definite, it has a unique square root $\tilde{\boldsymbol{K}}^{\frac{1}{2}}$. Thus $\tilde{\boldsymbol{K}}^{\frac{1}{2}}\boldsymbol{Py} \sim \mathcal{N}(\boldsymbol{0}, \sigma_\epsilon^2\tilde{\boldsymbol{K}}^{\frac{1}{2}}\boldsymbol{P}(\tilde{\boldsymbol{K}}^{\frac{1}{2}})^T)$. Since $\boldsymbol{P}$ is symmetric and idempotent, we have $\boldsymbol{P} = \boldsymbol{P}^2 = \boldsymbol{P}\boldsymbol{P}^{\mathrm{T}}$ so that:

$$\begin{aligned}\tilde{\boldsymbol{K}}^{\frac{1}{2}}\boldsymbol{P}(\tilde{\boldsymbol{K}}^{\frac{1}{2}})^T &= \tilde{\boldsymbol{K}}^{\frac{1}{2}}\boldsymbol{P}\boldsymbol{P}^T(\tilde{\boldsymbol{K}}^{\frac{1}{2}})^T \\ &= \left(\tilde{\boldsymbol{K}}^{\frac{1}{2}}\boldsymbol{P}\right)\left(\tilde{\boldsymbol{K}}^{\frac{1}{2}}\boldsymbol{P}\right)^T\end{aligned}$$

Thus, $\tilde{\boldsymbol{K}}^{\frac{1}{2}}\boldsymbol{P}(\tilde{\boldsymbol{K}}^{\frac{1}{2}})^T \succeq 0$ so that $\tilde{\boldsymbol{K}}^{\frac{1}{2}}\boldsymbol{P}(\tilde{\boldsymbol{K}}^{\frac{1}{2}})^T = \boldsymbol{U\Sigma U}^T$ where $\boldsymbol{\Sigma} = diag(\rho_1, \ldots, \rho_N)$ and $\boldsymbol{U}^T\boldsymbol{U} = \boldsymbol{I}$. Setting $\boldsymbol{B} = \boldsymbol{U}^T\tilde{\boldsymbol{K}}^{\frac{1}{2}}\boldsymbol{P}$, we have $\boldsymbol{By} \sim \mathcal{N}(\boldsymbol{0}, \sigma_\epsilon^2\boldsymbol{\Sigma})$. We then have:

$$\begin{aligned}Q &\equiv \frac{1}{\sigma_\epsilon^2}\boldsymbol{y}^T\boldsymbol{P}\tilde{\boldsymbol{K}}\boldsymbol{P}\boldsymbol{y} \\ &= \frac{1}{\sigma_\epsilon^2}(\boldsymbol{U}\tilde{\boldsymbol{K}}^{\frac{1}{2}}\boldsymbol{Py})^T(\boldsymbol{U}\tilde{\boldsymbol{K}}^{\frac{1}{2}}\boldsymbol{Py}) = \frac{1}{\sigma_\epsilon^2}(\boldsymbol{By})^T\boldsymbol{By} \sim \sum_{n=1}^{N}\rho_n\chi_1^2\end{aligned}$$

The eigenvalues of $\boldsymbol{K}^{\frac{1}{2}}\boldsymbol{P}(\boldsymbol{K}^{\frac{1}{2}})^T$ are equal to the eigenvalues of $\boldsymbol{U}^T\boldsymbol{K}^{\frac{1}{2}}\boldsymbol{PP}(\boldsymbol{K}^{\frac{1}{2}})^T\boldsymbol{U} = \boldsymbol{BB}^T$. Using the fact that for every matrix $\boldsymbol{B}$, $\boldsymbol{B}^T\boldsymbol{B}$ and $\boldsymbol{BB}^T$ have the same nonzero eigenvalues, we can equivalently calculate ρ_n using the nth eigenvalue of the matrix $\boldsymbol{P}\tilde{\boldsymbol{K}}\boldsymbol{P}$.

□
