# Supplementary material for: Fast kernel-based association testing of non-linear genetic effects for biobank-scale data
Source: Nat Commun. 2023 Aug 15;14:4936. doi: 10.1038/s41467-023-40346-2 (PMC10427662; doi:10.1038/s41467-023-40346-2)
Supplement: Supplementary file 3 — Reporting Summary [file 41467_2023_40346_MOESM3_ESM.pdf]

nature portfolio

Corresponding author(s): Sriram Sankararaman
Last updated by author(s): Jun 18, 2023

Reporting Summary

Nature Portfolio wishes to improve the reproducibility of the work that we publish. This form provides structure for consistency and transparency in reporting. For further information on Nature Portfolio policies, see our Editorial Policies and the Editorial Policy Checklist.

Statistics

For all statistical analyses, confirm that the following items are present in the figure legend, table legend, main text, or Methods section.

n/a	Confirmed	
☐	☒	The exact sample size (*n*) for each experimental group/condition, given as a discrete number and unit of measurement
☒	☐	A statement on whether measurements were taken from distinct samples or whether the same sample was measured repeatedly
☐	☒	The statistical test(s) used AND whether they are one- or two-sided<br>*Only common tests should be described solely by name; describe more complex techniques in the Methods section.*
☐	☒	A description of all covariates tested
☐	☒	A description of any assumptions or corrections, such as tests of normality and adjustment for multiple comparisons
☐	☒	A full description of the statistical parameters including central tendency (e.g. means) or other basic estimates (e.g. regression coefficient) AND variation (e.g. standard deviation) or associated estimates of uncertainty (e.g. confidence intervals)
☐	☒	For null hypothesis testing, the test statistic (e.g. *F*, *t*, *r*) with confidence intervals, effect sizes, degrees of freedom and *P* value noted<br>*Give P values as exact values whenever suitable.*
☒	☐	For Bayesian analysis, information on the choice of priors and Markov chain Monte Carlo settings
☐	☒	For hierarchical and complex designs, identification of the appropriate level for tests and full reporting of outcomes
☐	☒	Estimates of effect sizes (e.g. Cohen's *d*, Pearson's *r*), indicating how they were calculated

Our web collection on statistics for biologists contains articles on many of the points above.

Software and code

Policy information about availability of computer code

Data collection	No software was used
Data analysis	The code for FastKAST and data analysis are freely available at https://github.com/sriramlab/FastKAST; the simulator is freely available at https://github.com/alipazokit/simulator; SKAT software (v. 2.2.5) instruction can be found at at https://cran.r-project.org/web/packages/SKAT/index.html

For manuscripts utilizing custom algorithms or software that are central to the research but not yet described in published literature, software must be made available to editors and reviewers. We strongly encourage code deposition in a community repository (e.g. GitHub). See the Nature Portfolio guidelines for submitting code & software for further information.

Data

Policy information about availability of data

All manuscripts must include a data availability statement. This statement should provide the following information, where applicable:

- Accession codes, unique identifiers, or web links for publicly available datasets
- A description of any restrictions on data availability
- For clinical datasets or third party data, please ensure that the statement adheres to our policy

The UK Biobank dataset used in this study is not publicly available but can be obtained by application (https://www.ukbiobank.ac.uk/).

Research involving human participants, their data, or biological material

Policy information about studies with human participants or human data. See also policy information about sex, gender (identity/presentation), and sexual orientation and race, ethnicity and racism.

Reporting on sex and gender	We analyzed unrelated white British individuals from the UK Biobank data. Sex information has been treated as covariate and was regressed out in our model. Our findings are relevant to the general population in the UK Biobank and are not gender/sex specific.
Reporting on race, ethnicity, or other socially relevant groupings	N/A
Population characteristics	UK Biobank recruited individuals aged between 40 and 69 years with a roughly equal representation of men and women. We took the age, sex, and the top 20 PCs of the genotype matrix as our covariates.
Recruitment	Details can be found at UK Biobank website (https://www.ukbiobank.ac.uk/).
Ethics oversight	There was no protocol specific to our study. De-identified data from the UK Biobank was obtained through an application submitted by the PI to the UK Biobank (https://www.ukbiobank.ac.uk/. Application number 33127).

Note that full information on the approval of the study protocol must also be provided in the manuscript.

Field-specific reporting

Please select the one below that is the best fit for your research. If you are not sure, read the appropriate sections before making your selection.

☒ Life sciences ☐ Behavioural & social sciences ☐ Ecological, evolutionary & environmental sciences

For a reference copy of the document with all sections, see nature.com/documents/nr-reporting-summary-flat.pdf

Life sciences study design

All studies must disclose on these points even when the disclosure is negative.

Sample size	Individuals were selected by keeping unrelated white British individuals which are >3rd degree relatives (defined as pairs of individuals with kinship coefficient <1/2(9/2), computed using KING), and removing individuals with putative sex chromosome aneuploidy. This end up with 291,273 samples in total. This is standard sample-level QC that has been used in other published work from our lab (Pazokitoroudi et al. Nature Communications 2020) and other labs.
Data exclusions	Excluded individuals that were not classified as white British. Also removed closely-related individuals (closer than third-degree relatives)
Replication	There was no replication in our study.
Randomization	The study was designed by the UK Biobank. The goal of our paper was to analyze the collected data.
Blinding	Data was already collected by the UK Biobank so blinding is not applicable.

Reporting for specific materials, systems and methods

We require information from authors about some types of materials, experimental systems and methods used in many studies. Here, indicate whether each material, system or method listed is relevant to your study. If you are not sure if a list item applies to your research, read the appropriate section before selecting a response.

Materials & experimental systems

n/a	Involved in the study	
☒	☐	Antibodies
☒	☐	Eukaryotic cell lines
☒	☐	Palaeontology and archaeology
☒	☐	Animals and other organisms
☒	☐	Clinical data
☒	☐	Dual use research of concern
☒	☐	Plants

Methods

n/a	Involved in the study	
☒	☐	ChIP-seq
☒	☐	Flow cytometry
☒	☐	MRI-based neuroimaging
